# Qishen Granule Improved Cardiac Remodeling *via* Balancing M1 and M2 Macrophages

**DOI:** 10.3389/fphar.2019.01399

**Published:** 2019-11-25

**Authors:** Wenji Lu, Qiyan Wang, Xiaoqian Sun, Hao He, Qixin Wang, Yan Wu, Yue Liu, Yong Wang, Chun Li

**Affiliations:** ^1^College of Chinese Medicine, Beijing University of Chinese Medicine, Beijing, China; ^2^School of Life Sciences, Beijing University of Chinese Medicine, Beijing, China; ^3^Modern Research Center for Traditional Chinese Medicine, School of Chinese Materia Medica, Beijing University of Chinese Medicine, Beijing, China; ^4^Center of Scientific Experiment, Beijing University of Chinese Medicine, Beijing, China; ^5^Cardiovascular Disease Center, Xiyuan Hospital, China Academy of Chinese Medical Sciences, Beijing, China

**Keywords:** Qishen Granule, myocardial fibrosis, splenic monocytes, macrophages, angiogenesis

## Abstract

Macrophages play a pivotal role in myocardial remodeling (MR) process which could eventually lead to heart failure. Splenic monocytes could be mobilized and recruited under inflammatory conditions and differentiated into different types of macrophages in heart tissues. Inflammatory M1 macrophages could aggravate tissue damage whereas M2 macrophages could promote angiogenesis and tissue repair process. Unbalanced ratio of M1/M2 macrophages may eventually lead to adverse remodeling. Therefore, regulating differentiation and activities of macrophages are potential strategies for the management of myocardial remodeling. Qishen Granule (QSG) is an effective Chinese medicine for treating heart failure. Our previous studies demonstrated that QSG could inhibit myocardial fibrosis through regulating secretion of cytokines and activation of macrophages. However, the detailed effects of QSG on had not been elucidated yet. In this study, we aimed to explore the effect of QSG on the release of splenic monocytes, the recruitment of monocytes into heart tissues and the differentiation of macrophages under ischemic conditions. Our results showed that QSG could suppress the release of monocytes from the spleen and recruitment of monocytes to heart tissues *via* inhibiting splenic angiotensin (Ang) II/AT1-cardiac monocyte chemotactic protein (MCP)-1/CC chemokine receptor 2 (CCR2) pathway. The anti-fibrotic effect of QSG was exerted by inhibiting M1 macrophage-activated transforming growth factor (TGF)-β1/Smad3 pathway. Meanwhile, QSG could promote angiogenesis by promoting differentiation of M1 macrophages into M2 macrophages. Our results suggest that compounds of Chinese medicine have synergistic effects on cardiac and splenic organs through regulating differentiation of monocytes/macrophages in inhibiting myocardial remodeling.

## Introduction

Myocardial remodeling (MR) is an important pathophysiological change after myocardial infarction, and is a common pathological change during the development of heart failure (HF) (Pryds et al., 2019). Macrophage-mediated inflammation contributes greatly to fibrosis and prolonged inflammation will eventually lead to MR. Macrophages also participate in the healing process after myocardial infarction. Therefore, regulating differentiation and activities of macrophages are potential strategies for the management of MR to prevent HF (Honold and Nahrendorf, 2018).

The myocardium is mainly composed of cardiomyocytes, fibroblasts, endothelial cells and macrophages (Hu et al., 2017). Macrophages are involved in myocardial injury, repair and remodeling processes during the inflammatory response (Hulsmans et al., 2018). In steady state, most cardiac macrophages are derived from local progenitors and their functions remain largely unknown. It is speculated that these resident macrophages may play roles in guarding against infection, regulating angiogenesis and directing matrix turnover (Jablonski et al., 2015). Under inflammatory condition, such as myocardial infarction, circulating monocytes will be abundantly recruited to ischemic heart tissues through monocyte chemotactic protein-1 (MCP-1)/CC chemokine receptor 2 (CCR2) interactions. Once recruited, monocytes can differentiate into different types of macrophages which participate either in inflammatory or repairing process. According to surface markers and functions, macrophages can be roughly classified into inflammatory M1 and less inflammatory M2 types. M1 macrophages participate in inflammatory response by secreting pro-inflammatory cytokines interleukin (IL)-6 and chemokines, which aggravate tissue damage (Momtazi-Borojeni et al., 2019). M2 macrophages have only weak antigen-presenting ability, and they secrete vascular endothelial growth factor (VEGF) which promotes angiogenesis and tissue repair (Wu et al., 2010). The dynamic regulation and balance between M1 and M2 macrophages will determine the ventricular remodeling process.

The spleen is the largest organ in the immune system. Besides, it is a huge reservoir of more than half of the body’s monocytes (Halade et al., 2018). About 40–70% of monocytes that are recruited to myocardial infarct site are derived from the spleen (Sager et al., 2017). The resting spleen contains a large reservoir of bona fide monocytes (Robbins and Swirski, 2010). Under inflammatory condition, angiotensin (Ang) II activates the Ang II type I receptor (AT1) in the spleen, allowing a large number of monocytes to be released into the blood. The role that spleen plays in myocardial infarction remains to be fully investigated. Inhibiting release of monocytes from spleen could reduce the number of macrophages in heart tissues and ameliorate inflammation (Partha D. Nahrendorf, 2015). Therefore, spleen is a potential target in the management of MR.

Qishen Granule (QSG) is prepared from the combination of the classic prescription Zhenwu Tang and Si Miao Yong An Tang in traditional Chinese medicine (Li et al., 2014). QSG mainly consists of six Chinese herbs including *A. membranaceus* (Fisch.) Bunge., *S. Miltiorrhiza* Bunge., *L. japonica* Thunb., *S. aestivalis* Griseb., *A. fischeri* Rchb. and *G. uralensis* Fisch (Li et al., 2016). The chemical component of QSG has been verified by liquid chromatography-mass spectrometry techniques (Guo et al., 2016). Preclinical studies have proven that QSG can effectively improve cardiac function and inhibit myocardial fibrosis (Wang et al., 2019). It has also been reported that QSG could protect against myocardial damage after macrophage activation (Liu et al., 2016). However, it hasn’t been clarified if QSG could inhibit activation of macrophages through inhibiting release of monocytes from spleen. The regulative effect of QSG on differentiation of macrophages is also unclear. In this study, we aimed to explore if QSG could ameliorate myocardial remodeling through suppressing release of monocytes from spleen and inhibiting macrophage recruitment in a HF rat model. The roles of QSG on macrophage differentiation and splenic AngII/AT1-cardiac MCP-1/CCR2 pathway were investigated. This study will provide alternative targets in the management of macrophage-induced myocardial remodeling.

## Materials and Methods

### Animals

All animal experimental protocols were approved by the Ethics Committee of Beijing University of Chinese Medicine and conformed to the Guide for the Care and Use of Laboratory Animals published by the U.S. National Institute of Health (NIH Publication No. 85-23, revised 1996). 60 male Sprague–Dawley (SD) healthy rats (220 ± 20 g) in Specific Pathogen Free (SPF) grade used in this study were obtained from Beijing Vital River Laboratory Animal Technology Co. Ltd (China). The rats were housed in the cages under standard laboratory conditions (12 h light/dark cycle, controlled temperature of 25°C).

### Drugs

Qishen Qranule is composed of *A. membranaceus* (Fisch.) Bunge., *S. Miltiorrhiza* Bunge., *L. japonica* Thunb., *S. aestivalis* Griseb., *A. fischeri* Rchb. and *G. uralensis* Fisch, purchased from Beijing Tongrentang (Group) Co., Ltd. and all the authentication of plant materials was identified by Dr. Jian Ni at Beijing University of Chinese Medicine. The voucher specimens (Voucher numbers: *A. membranaceus* (Fisch.) Bunge-2016-007; *S. Miltiorrhiza* Bunge-2016-008; *L. japonica* Thunb-2016-009; *S. aestivalis* Griseb-2016-010; *A. fischeri* Rchb-2016-011; *G. uralensis* Fisch-2016-012) were submitted to Department of Chinese medicine teaching and Research, School of Traditional Chinese Medicine, Beijing University of Chinese Medicine (Wang et al., 2017). The previous team tested the chemical composition of QSG by Ultra-high performance liquid chromatography coupled with hybridion trap-time of flight mass spectrometry (UHPLC-IT-TOF-MS), and the results showed that Glycyrrhizic acid, Formononetin, Dihydrotanshinone I, Cryptotanshinone, Tanshinone I, Tanshinone I, Glycyrrhetic acid, and Tanshinone IIA were the main component (Guo et al., 2016). The fingerprint of QSG was analyzed by HPLC and the typical chromatograms were shown in **Supplementary Data 1**. Fosinopril was provided by Sino-US Shanghai Squibb Pharmaceutical Co. Ltd. Country Medicine Accurate Character Number: H19980197.

### Animal Model of Heart Failure and Drug Administration

Left anterior descending (LAD) coronary artery of rats was ligated to induce the HF model as described in our previous studies (Wang et al., 2013). Briefly, 60 SD rats underwent left thoracotomy between the third and fourth intercostal space. After exposure of heart tissues, the LAD was ligated with a sterile suture 1 mm below the left atrium in 50 rats. Rats in sham group only underwent thoracotomy and threading in the same position of the heart. Ten rats that underwent LAD ligation were randomly chosen for splenectomy surgery using previously published method (Schwarz and Hiserodt. 1990). Approximately 1 cm incision was made by midline laparotomy, hepatic hilum was clamped, spleen was removed, and the muscles and skin were sutured with silk suture.

The main causes of mortality in myocardial infarction rats were anesthesia, laryngeal edema, excessive blood loss, arrhythmia, lung injury, pneumothorax, etc (Degabriele et al., 2004). Within 1 day after surgery, 8 rats died (1 rat in the splenectomy group) and 52 survived. The survival rate was 86.7%. 52 rats were randomly divided into five groups: 10 rats in the sham group, 11 rats in the model group, 9 rats in the splenectomy group, 11 rats in the QSG group, and 11 rats in the fosinopril group.

Rats in QSG group were treated with QSG at the dosage of 235.2 mg/kg per day and rats in fosinopril group were treated with fosinopril at the dosage of 4.67 mg/kg per day for 21 d. All the drugs were gavaged with the amount of l ml/100 g. Rats in the sham group, model group and splenectomy group were administrated with the same volume of water for 21 d.

### Evaluation of Echocardiographic Parameters

21 d after treatment, rats inhaled anesthetic isoflurane and vevo 2100 was applied to observe the echocardiographic parameters of rats to assess the cardiac functions (Vevo TM 2100, Visual Sonics, Canada). Short axis was used to measure the length of left ventricular anterior wall; systole (LVAW;s), left ventricular anterior wall; diastole (LVAW;d), left ventricular internal diameter; diastole (LVID;d), left ventricular internal diameter; systole (LVID;s), left ventricular posterior wall; systole (LVPW;s), left ventricular posterior wall; diastole (LVPW;d). The values of ejection fraction (EF) and fractional shortening (FS) were calculated according to the following equation: FS% = [(LVID;d − LVID;s)/LVID;d] × 100%; EF% = [(LVEDV − LVESV)/LVEDV] × 100%.

### Histological Examination

The heart and spleen tissues were fixed in 4% paraformaldehyde for 48 h, and then embedded in paraffin and cut into 5 µm sections. The spleen tissues were stained with hematoxylin–eosin (HE) staining to assess the basic structure. The heart tissues were stained with Masson staining and Sirius red staining to assess the extent of inflammatory infiltration and myocardial fibrosis.

### Measurement of angiotensin II in the serum by radioimmunoassay

The level of angiotensin II was quantified directly from prepared plasma using radioimmunoassay (RIA) kits (Beijing Sino-UK institute of biological technology, Beijing, China) according to protocol of the manufacturer.

### Measurement of Indicators by Immunohistochemistry

The heart sections were deparaffinized in xylene, rehydrated in alcohol gradient and then rinsed in PBS. The samples were incubated with primary antibody (Anti-CD31, GB12063, Servicebio, China) for 12 h at 4°C after being blocked with 0.3% hydrogen peroxide, avidin biotin blocking reagent, and biotin blocking reagent for 15 min. The slides were then incubated with the biotinylated secondary antibody for 2 h and the stained with hematoxylin for 30 s. The slides were then dehydrated by graded ethanol and xylene and fixed with resin glue. The positive area was specifically stained brown-yellow.

### Measurement of Indicators by Immunofluorescence

The sections were sequentially dewaxed in xylene I and II, rehydrated in an alcohol gradient, and then placed in a citrate antigen retrieval buffer (pH 8.0) for antigen retrieval. Normal serum was added to the slides and blocked at room temperature for 30 min. After blocking, sections were incubated overnight with anti-CD68 (ab201340, Abcam, United States), anti-CD86 (ab53004, Abcam, United States), anti-CD163 (ab182422, Abcam, United States) primary antibody at 4°C and with fluorescent secondary antibody for 1 h at 37°C. The sections were further stained with DAPI staining for 5 min in the dark. Finally, the sections were sealed on cover slips and fluorescence images were captured using fluorescence microscope.

### Measurement of Indicators by Western Blot

Tissue protein of heart and spleen was lysed by the RIPA lysate (50:1) and tissue disrupter and quantified by the BCA method. The quantified homogenate was added to loading buffer, and the mixture was boiled and denatured at 99°C. Each lane was loaded with 10 ul of proteins. After electrophoresis at 100 V for 1–1.5 h, proteins were transferred to PVDF membranes at 300 mA for 1–1.5 h. Afterwards, the membrane blocked with 5% skim milk for 1–1.5 h at room temperature, incubated on a shaker, and washed with TBS-T. Western blot analysis was conducted using anti-AT1 (ab18801; Abcam, United States), anti-MCP-1 (ab25124, Abcam, United States), anti-CCR2 (PAI-27409), anti-TGF-β1 (3711s, Cell Signaling Technology, Germany), anti-Smad3 (ab28379, Abcam, United States), anti-MMP2 (ab86607, Abcam, United States), anti-Col III (ab7778, Abcam, United States), anti-VEGF (ab10972, Abcam, United States), anti-CD31 (ab24590, Abcam, United States) and anti-GAPDH (ab8245, Abcam, United States) at 4°C overnight. After incubation with the appropriate secondary antibodies at 37°C for 2 h at room temperature, the membrane treated with ECL for 1 min at room temperature. The final expression of each protein was normalized by GAPDH and grayscale analysis was performed using Image J software.

### Statistical Analysis

All data were expressed as mean ± standard deviation (SD). One-way analysis of variance (ANOVA) and Dunnett’s test were used to compare differences among multiple groups. The values of *P* < 0.05 were considered as statistically significant. All analyses were performed using SPSS 17.0 or GraphPad Prism 7.

## Results

### QSG Inhibited the Release of Monocytes From the Spleen

**Figure 1:**
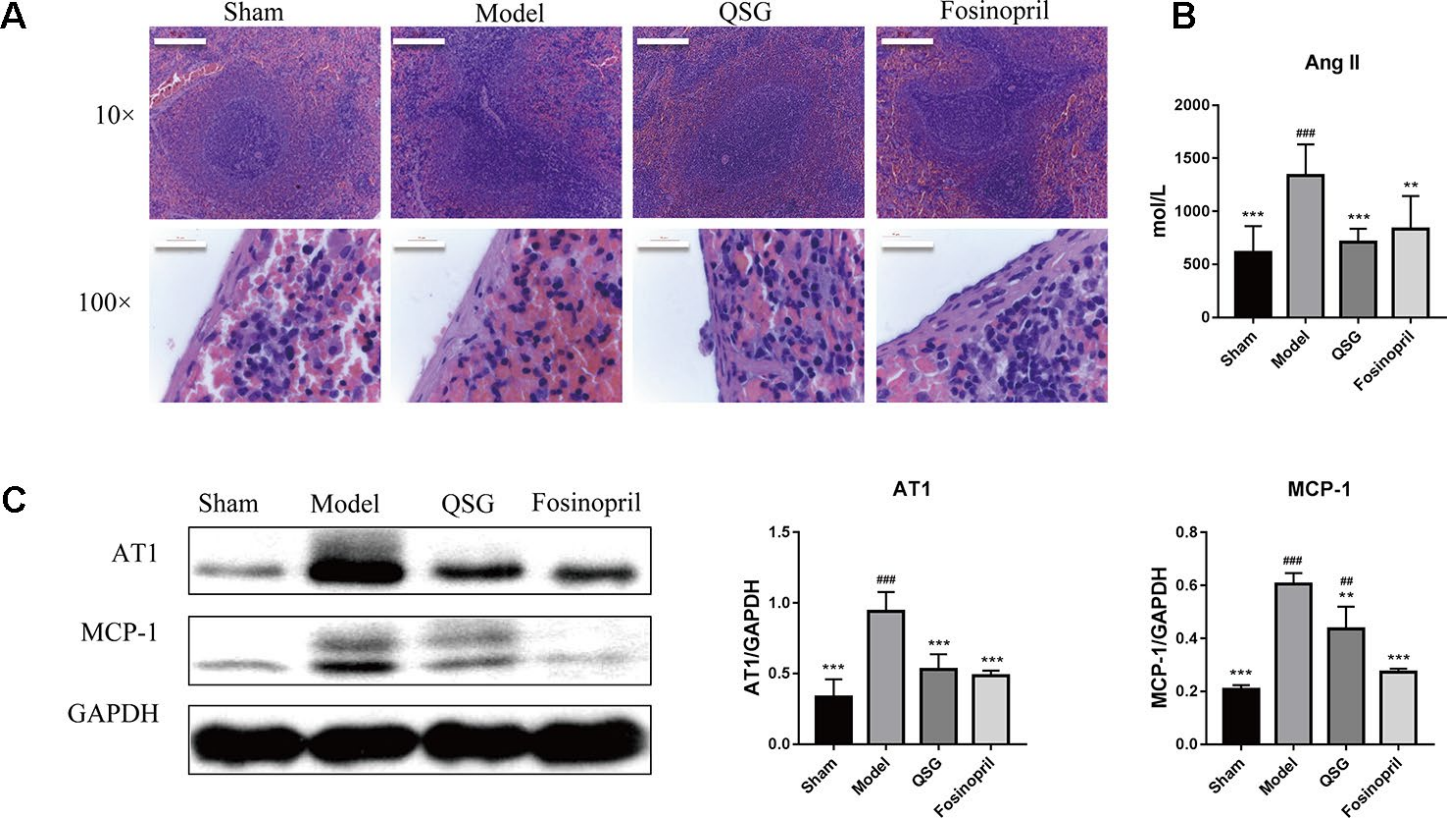
QSG inhibited the release of monocytes from the spleen. **(A)** HE staining showed that monocytes in the marginal zone of the spleen were released in the model group, and QSG could exert the protective effect by increasing the number of monocytes in red pulp under the capsule and marginal zone of the spleen. **(B)**. QSG significantly decreased the circulatory level of angiotensin II. **(C)** Western blot showed that QSG reduced the expressions of AT1 and MCP-1 in splenic tissues. All data were presented as means ± SD from independent experiments performed in triplicate. ^##^ *P* < 0.01, ^###^ *P* < 0.001 vs the sham group; **P < 0.01, ***P < 0.001 vs the model group. N = 3 per group.

Heart failure results in increased motility of monocytes in the spleen (Swirski FK et al., 2009). First, we assessed the number of monocyte in the spleen on 21 d after LAD ligation. The subcapsular region is the site where monocytes are stored in the spleen. HE staining showed that the number of monocytes in the marginal zone of the spleen was decreased after LAD ligation compared with the sham group, indicating that after LAD ligation, spleen released a large number of monocytes. Second, the effect of QSG on the release of monocytes from spleen was investigated. QSG could significantly increase the number of monocytes in red pulp under the capsule and marginal zone of the spleen as compared with those in the model group (**Figure 1A**). Fosinopril, an angiotensin-converting enzyme inhibitor (ACEI), was applied as the positive control drug. Circulatory level of angiotensin II in model group was increased compared with sham group. Consistently, levels of AT1 and MCP-1 in the spleen were also up-regulated in the model group. Treatment of QSG and fosinopril could significantly down-regulate the levels of AngII, AT1 and MCP-1, indicating that QSG could suppress the release of monocytes from the spleen *via* inhibiting Ang II/AT1 signaling pathway (**Figures 1B, C**).

### QSG Inhibited Recruitment of Monocytes and M1 Macrophages-Induced Activation of Transforming Growth Factor (TGF)-B1/Smad3 in the Border Zone of Infarcted Heart

**Figure 2:**
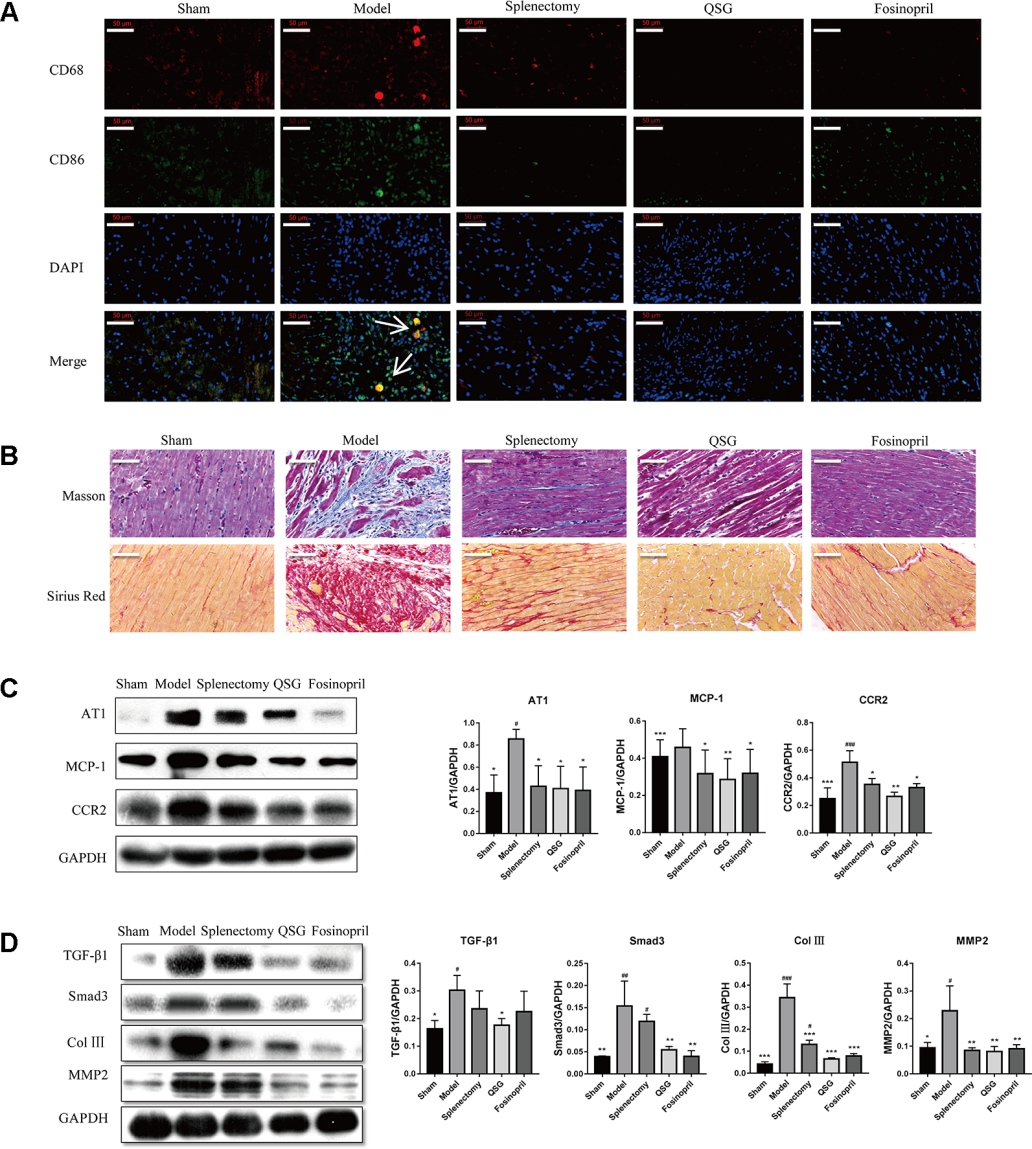
QSG inhibited activation of TGF-β1/Smad3 through reducing the recruitment of M1 macrophages in myocardial tissue. **(A)** IF staining showed that splenectomy reduced the recruitment of M1 macrophages in myocardial tissue; QSG treatment decreased the recruitment of M1 macrophages. **(B)** Masson and Sirius Red staining showed that QSG inhibited cardiac fibrosis. **(C)** Western blot showed that QSG down-regulated the expressions of AT1, MCP-1 and CCR2 in myocardial tissue compared with that in the model group. **(D)** Western blot showed that QSG decreased the expressions of TGF-β1, Smad3, collagen III and MMP2 in cardiac tissue. All data were presented as means ± SD from independent experiments performed in triplicate. ^#^ *P* < 0.05, ^##^ *P* < 0.01, ^###^ *P* < 0.001 vs the sham group; *P < 0.05, **P < 0.01, ***P < 0.001 vs the model group. N = 3 per group.

The effect of QSG on the recruitment of monocytes to infarcted heart tissues was further examined. Immunofluorescence results showed that the number of M1 macrophages (CD68 + CD86 positive cells) increased significantly in the model group relative to the sham group. QSG could reduce the number of M1 macrophages in the border zone of infarcted heart as compared with the model group. Splenectomy was applied to investigate whether the increased macrophages in heart were originated from the spleen. After splenectomy, the number of M1 macrophages was reduced significantly (**Figure 2A**). Splenectomy could also significantly alleviate inflammation and fibrosis in the heart tissues, demonstrating that splenic monocytes played important role in myocardial remodeling (**Figure 2B**). Since AT1 receptor-activated MCP-1/CCR2 interaction is critically involved in the recruitment of monocytes to heart tissues (França et al., 2017), the effects of QSG on the expressions of AT1, MCP-1 and CCR2 were then examined. Results showed that the levels of AT1, MCP-1 and CCR2 were significantly increased in the model group, as compared with the sham group. After treatment with QSG, expressions of AT1, MCP-1 and CCR2 were down-regulated, indicating that QSG could inhibit recruitment of monocytes through MCP-1/CCR2 interactions. Splenectomy and fosinopril had similar effects as QSG treatment (**Figure 2C**).

When monocytes are recruited to heart tissues, they could differentiate into M1 macrophages, which are able to stimulate production of TGF-β1 by fibroblasts, activate Smad3 and induce production of collagens and MMPs, thereby promoting fibrosis and myocardial remodeling (Shivshankar et al., 2014). Western blot showed that QSG could down-regulate expressions of TGF-β1 and Smad3 in the border zone of myocardial infarction. Levels of collagen III and matrix metallopeptidase 2 (MMP2) were also reduced by QSG treatment. Splenectomy and fosinopril had similar effects (**Figure 2D**). These results suggested that QSG could ameliorate myocardial remodeling by inhibiting macrophage-activated TGF-β1/Smad3 pathway.

### QSG Induced Differentiation of M2 Macrophages and Promoted Angiogenesis in Myocardial Tissue

**Figure 3:**
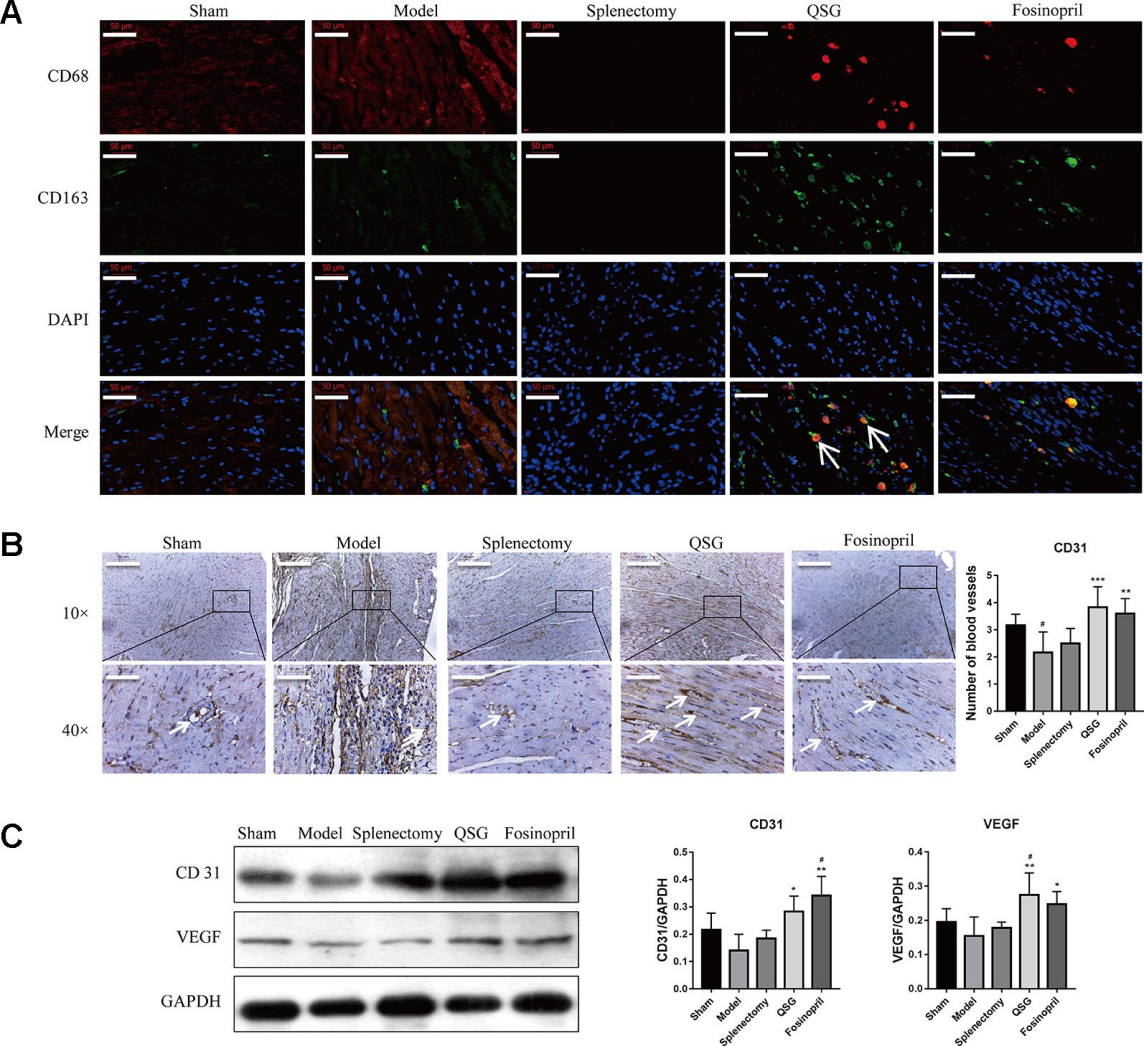
QSG promoted angiogenesis through inducing the number of M2 macrophages in myocardial tissue. **(A)** IF staining showed that QSG treatment up-regulated the M2 macrophages. After splenectomy, the number of M2 macrophages remained unchanged relative to the model group. **(B)** CD31 staining showed that QSG could promote angiogenesis. **(C)** Western blot showed that QSG increased the expressions of CD31 and VEGF in myocardial tissue compared with model group. All data were presented as means ± SD from independent experiments performed in triplicate. ^#^ *P* < 0.05 vs the sham group; *P < 0.05, **P < 0.01, ***P < 0.001 vs the model group. N = 3 per group.

**Figure 4:**
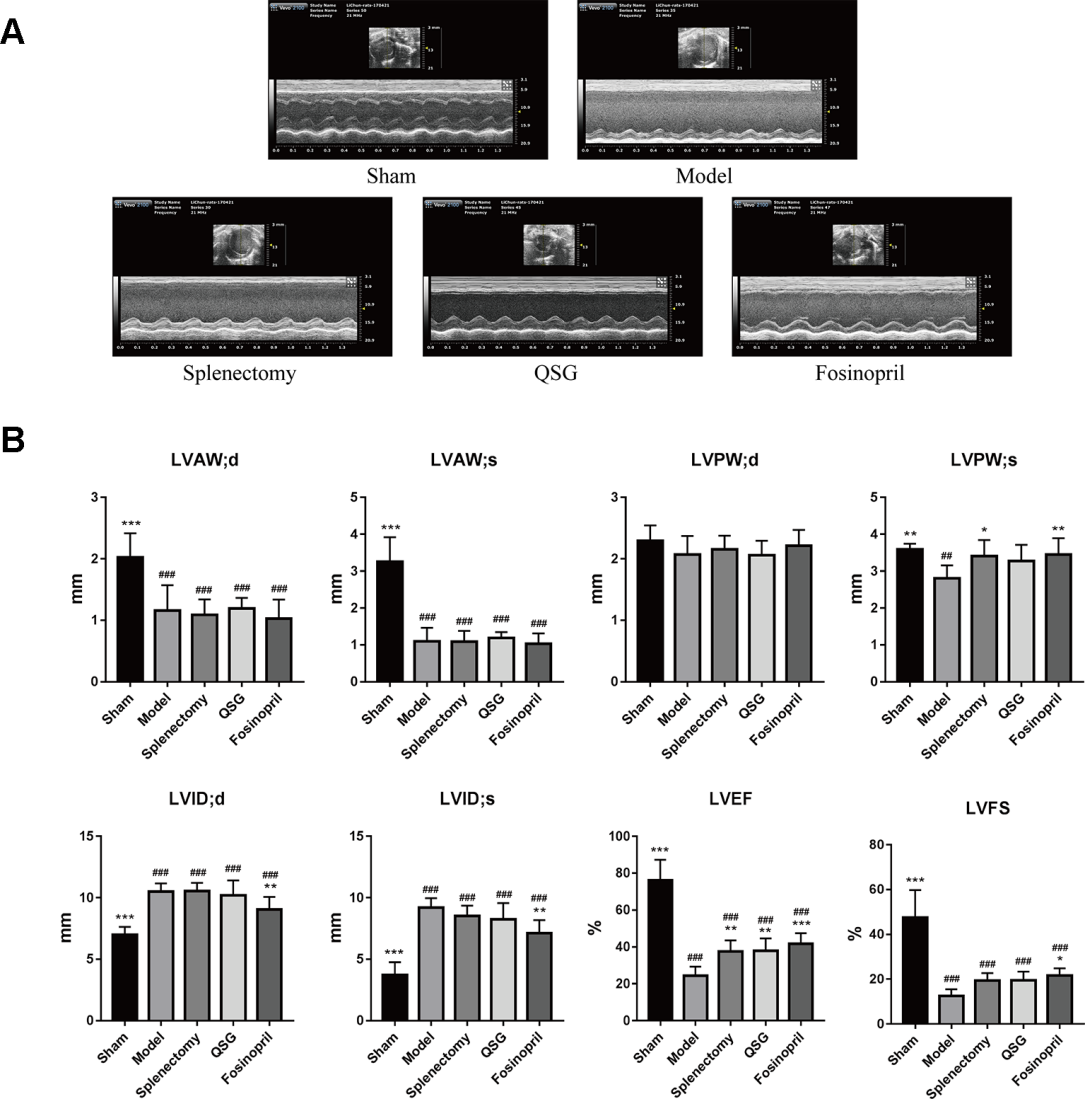
QSG improves cardiac function by echocardiography. **(A)** Representative images of echocardiography exhibiting the changes in cardiac function in each group. **(B)** Echocardiography results showed that QSG and splenectomy improved left ventricular ejection fraction, and cardiac function of HF rats. ^##^ *P* < 0.01, ^###^ *P* < 0.001 vs the sham group; *P < 0.05, **P < 0.01, ***P < 0.001 vs the model group. N = 7 per group.

In contrast to M1 macrophages, M2 macrophages are involved in inflammation suppression, tissue repair and angiogenesis (Ribeiro et al., 2018). Therefore, the effect of QSG on differentiation of M2 macrophages was further investigated. Immunofluorescence staining (labeled by CD86 and CD163) showed that the number of M2 macrophages in QSG group was increased compared with model group. In splenectomy group, there were no detectable M2 macrophages (**Figure 3A**). Immunohistochemical staining of CD31 showed that the number of microvessels was significantly up-regulated after treatment with QSG. However, the effect of splenectomy group was less significant than of QSG group (**Figure 3B**). Western blot showed that compared with the sham group, the expression levels of VEGF and CD31 were significantly down-regulated in the model group. After treatments with QSG, the expression levels of VEGF and CD31 were increased (**Figure 3C**), demonstrating that QSG might promote angiogenesis through M2 macrophages. Fosinopril had similar effect with QSG. Echocardiography showed that compared with the sham group, left ventricular ejection fraction (EF) and fractional shortening (FS) of the model group were significantly decreased (*P* < 0.001). QSG increased EF by 56%, demonstrating that QSG could improve the left ventricular contractility. Splenectomy and fosinopril could also improve heart function (**Figure 4**).

## Discussion

Myocardial remodeling occurs in a variety of cardiovascular diseases and can be caused by inflammatory response, which is closely related to infiltration of macrophages (Frantz and Nahrendorf, 2014). Macrophage is an important cell population in healthy heart tissues. Macrophages have multiple phenotypes and functions. It is speculated that macrophages guard against infection and regulate matrix turnover under steady state, although the source of macrophages in healthy heart remains unclear (Davies and Taylor. 2015). Under inflammatory conditions, such as acute infarction, circulatory monocytes will be recruited to heart tissues and differentiate into different types of macrophages. These macrophages play critical roles in inflammation, fibrosis, angiogenesis and myocardial remodeling. Therefore, regulating the recruitment of monocytes and differentiation of macrophages is considered to be promising target for managing myocardial remodeling. In this study, we explored the effect of traditional Chinese medicine QSG on monocytes/macrophages in infarct heart and spleen tissues and investigated the mechanism by which QSG inhibited fibrosis and myocardial remodeling.

A rat model of LAD ligation was induced in this study. LAD ligation led to myocardial infarction, remodeling and eventual heart failure. We firstly explored the source of macrophages in the border zone of infarction site. In addition to bone marrow, the spleen is a reservoir of monocytes and may supply large amount of monocytes that can be recruited to heart tissues under inflammatory conditions. Studies have shown that 40–70% of the macrophages in myocardial infarct site are recruited from spleen (Okizaki et al., 2015). Some experiments have demonstrated that removal of the spleen could inhibit the release of splenic monocytes and protect heart function (Pinto et al., 2012). Therefore, we performed splenectomy to explore the role of splenic monocytes under myocardial infarction conditions. It was shown that 21 d after LAD ligation, there were infiltrations of M1 macrophages (inflammatory type) in heart tissues. Myocardial fibrosis and compromised heart function were also observed. Furthermore, the number of monocytes in the spleen was significantly reduced in the model group. After removal of spleen, the number of M1 macrophages in myocardial tissue was significantly reduced and fibrosis was attenuated. Heart function was also improved by splenectomy. These results demonstrate that spleen is an important source of M1 macrophages after occurrence of myocardial infarction. Inhibition of release of splenic monocytes could attenuate fibrosis and protect against heart failure.

Furthermore, release of splenic monocytes is dependent on activation of renin-angiotensin-aldosterone system (RAAS). Angiotensin II activates release of splenic monocytes through AT1 receptor, and ACE inhibitor can attenuate release of monocytes from spleen (Leuschner et al.2010). Our previous studies have indicated that QSG has anti-inflammatory effects (Wang et al, 2015). Therefore, the effect of QSG on release of splenic monocytes was investigated in current study. Impressively, we found that QSG treatment could inhibit release of monocytes from spleen in rats with LAD ligation, and circulatory level of angiotensin II was also reduced accompanied by the suppression of AT1 and MCP-1. These data suggest that QSG exerts anti-inflammatory effect by suppressing release of splenic monocytes through the AT1-MCP-1 pathway in spleen. Under myocardial infarction conditions, the monocytes released by spleen will be recruited to heart through MCP-1/CCR2 interaction. In heart tissues, monocytes will be differentiated into inflammatory M1 macrophages and alternative activated M2 macrophages (Moore and Tabas. 2011). M1 macrophages contribute to myocardial fibrosis by stimulating production of TGF-β1, which contributes to tissue fibrosis by stimulating Smads signaling and releasing extracellular matrix from myocardial myofibroblasts (Zhang et al., 2016). In our study, we found that QSG could reduce the number of M1 macrophages in the border zone of myocardial infarction and inhibit deposition of collagens. The expressions of AT1, MCP-1 and CCR2 in heart tissues were reduced accordingly. Furthermore, the levels of TGF-β1 and Smad3 were also suppressed. ACE inhibitor had similar effect as those of QSG. These data indicate that QSG may inhibit monocyte recruitment *via* MCP-1/CCR2 interaction and suppress M1 macrophage-induced fibrosis *via* TGF-β1/Smad3 pathway.

In addition, studies have shown that M1 macrophages are inflammatory whereas M2 macrophages participate in tissue repair, angiogenesis and resolution of inflammation (Liao et al., 2018). Promoting differentiation of monocytes into M2 macrophages might be beneficial for heart function (Singla et al, 2017). In this study, we observed that the number of M2 macrophages was up-regulated by QSG treatment. Intriguingly, there were no M2 macrophages observed in heart tissues in rats with splenectomy, as the reservoirs of monocytes, the spleen had been removed. Previous studies showed that M2 macrophages could promote angiogenesis by excreting VEGF (Oka et al., 2014). We found that the number of microvessels was significantly up-regulated after QSG treatment and the expressions of CD31 and VEGF were also increased by QSG. These results demonstrate that QSG could exert angiogenic effects through up-regulating differentiation of M2 macrophages.

**Figure 5:**
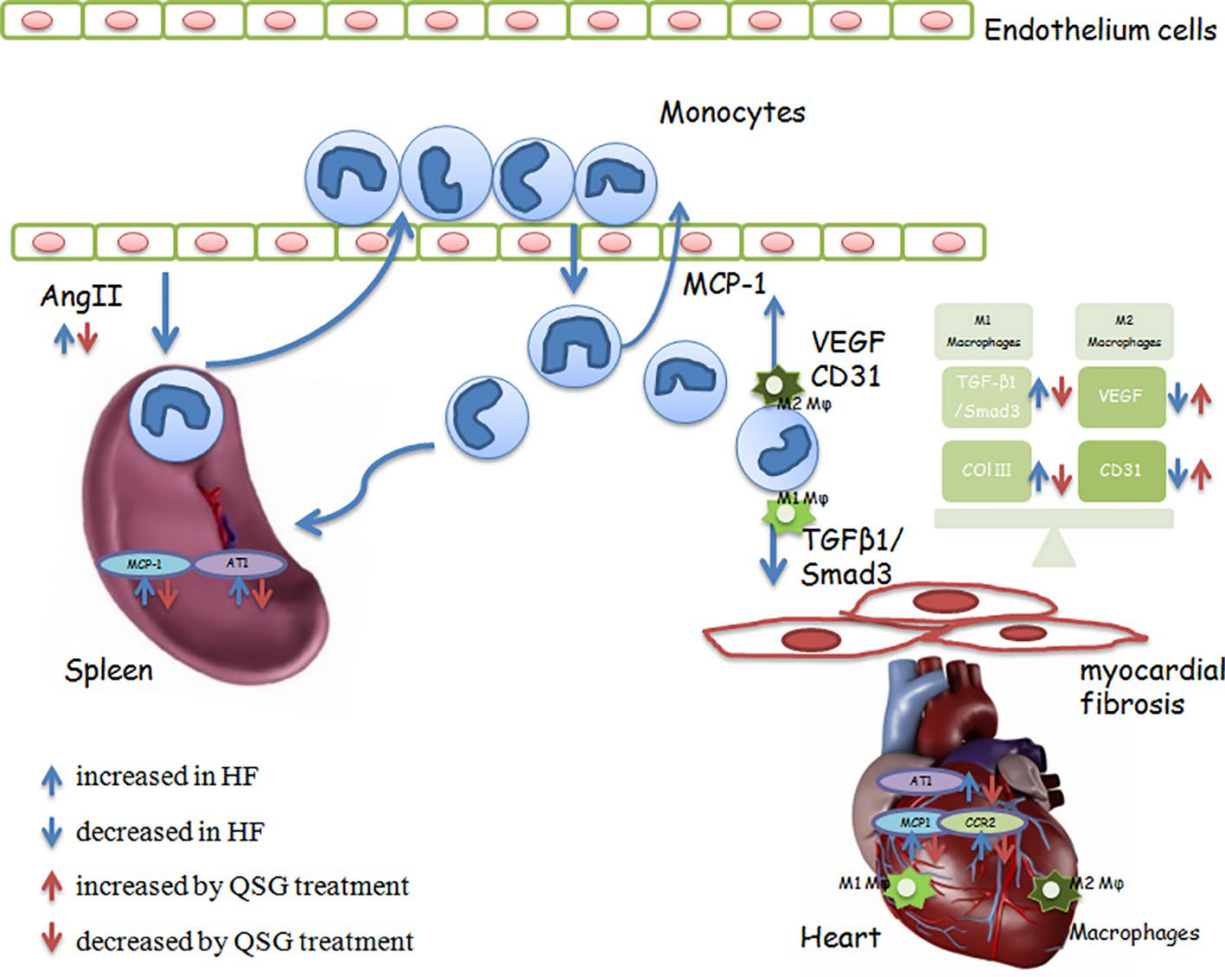
QSG suppresses the release of spleen monocytes and recruitment to heart tissues through the splenic Ang II/AT1-cardiac MCP-1/CCR2 pathway to inhibit MR.

In conclusion, QSG attenuated myocardial fibrosis through restoring the unbalance of M1 and M2 macrophages in heart tissues. Monocytes released by spleen could be recruited to heart tissues while QSG could suppress splenic release of monocytes. QSG could inhibit M1 macrophages-activated TGF-β1/Smad3 pathway and promote M2 macrophage-dependent angiogenesis (**Figure 5**). This study provides evidence that compounds of Chinese medicine have synergistic effect on cardiac and splenic organs. Moreover, restoring the balance of M1 and M2 macrophages can be a promising strategy for the management of myocardial remodeling.

## Data Availability Statement

All datasets generated for this study are included in the article/**Supplementary Material**.

## Ethics Statement

All animal experimental protocols were approved by the Ethics Committee of Beijing University of Chinese Medicine and conformed to the Guide for the Care and Use of Laboratory Animals published by the U.S. National Institute of Health (NIH Publication No. 85-23, revised 1996).

## Author Contributions

CL, YWa and YL designed the study. WL and QiyW did majority of experimental work and interpretation throughout the experiments. XS, HH and QixW analyzed the data. YWu operated cardiac ultrasound. All authors read and approved the final manuscript.

## Funding

This work was supported by the Grants from the National Natural Science Foundation of China (Nos. 81673802, 81530100, 81673712, and 81822049), Fok Ying Tung Education Foundation (No. 151044), Beijing Nova program (No. Z171100001117028), Talent Young Scientist of China Association for Science and Technology (No. CACM-2017-QNRC2-C13, No. CACM-2018-QNRC2-C07), the Fundamental Research Funds for the Central Universities (2017-JYB-JS-020).

## Conflict of Interest

The authors declare that the research was conducted in the absence of any commercial or financial relationships that could be construed as a potential conflict of interest.

## Abbreviations

Ang II, angiotensin II; CCR2, CC chemokine receptor 2; EF, ejection fraction; FS, fractional shortening; HF, heart failure; IL-6, Interleukin-6; LAD, left anterior descending; LVAW;s, left ventricular anterior wall; systole; LVAW;d, left ventricular anterior wall; diastole; LVID;d, left ventricular internal diameter; diastole; LVID;s, left ventricular internal diameter; systole; LVPW;s, left ventricular posterior wall; systole; LVPW;d, left ventricular posterior wall; diastole; MR, myocardial remodeling; MCP-1, monocyte chemotactic protein-1; MMP2, Matrix metallopeptidase 2; QSG, Qishen Granule; RAAS, renin angiotensin aldosterone system; TGF-β1, transforming growth factor-β1; VEGF, vascular endothelial growth factor.
