# Supplementary material for: Qishen Granule Improved Cardiac Remodeling via Balancing M1 and M2 Macrophages
Source: Front Pharmacol. 2019 Nov 25;10:1399. doi: 10.3389/fphar.2019.01399 (PMC6886583; doi:10.3389/fphar.2019.01399)
Supplement: Supplementary file 1 [file DataSheet_1.docx]

**SUPPLEMENTARY DATA 1**

HPLC-grade acetonitrile is product of Fisher Scientific (FairLawn, NJ, USA). Formic acid is product of Sigma Aldrich (St. Louis, MO, USA). Ultrapure water was prepared in our own laboratory by Milli-Q plus System (Millipore, Bedford, MA, USA). Analytical-grade solvents used for sample preparation are products of Beijing Chemical Factory (Beijing, China).

**Sample preparation for analysis**

QSG was weighed accurately (0.04 g) and placed into a 1.5 mL centrifuge tube containing 1.0 mL 50% aqueous methanol for 5 minutes in a vortex. Following centrifugation at 12000 rpm for 10 minutes in a centrifuge (Eppendorf, Melbourne, Australia). A supernatant (200 µL) was 5-fold diluted with 50% aqueous methanol and centrifuged at 12000 rpm for 10 minutes.

**HPLC conditions for chemical profile**

The HPLC analysis was carried out on a Shimadzu HPLC (two LC-20AD_XR_ solvent delivery units，a SIL-20AC_XR_ auto-sampler, a CTO-20AC column oven, a SPD-M20A PDA detector, a DGU-20A_3R_ degasser, and a CBM-20A controller). The chromatographic separation was performed on a Shiseido C_18_ column (150 × 2.1mm, 2.7 μm) at 35 ºC. 0.02% aqueous formic acid (A) and acetonitrile containing 0.02% formic acid (B) –were used as the mobile phase for analysis. The flow rate was set at 0.4 mL/min. The elution condition was applied with a gradient program as follows: 0–20 min, 2-18% B; 20–30 min, 18–30% B; 30–35 min, 30–45% B; 35–40 min, 45–65% B; 40–55 min, 65–95% B; 55–60 min, 95% B. 10 μL were injected into HPLC system for analysis. The typical chromatograms are shown in Fig. 1


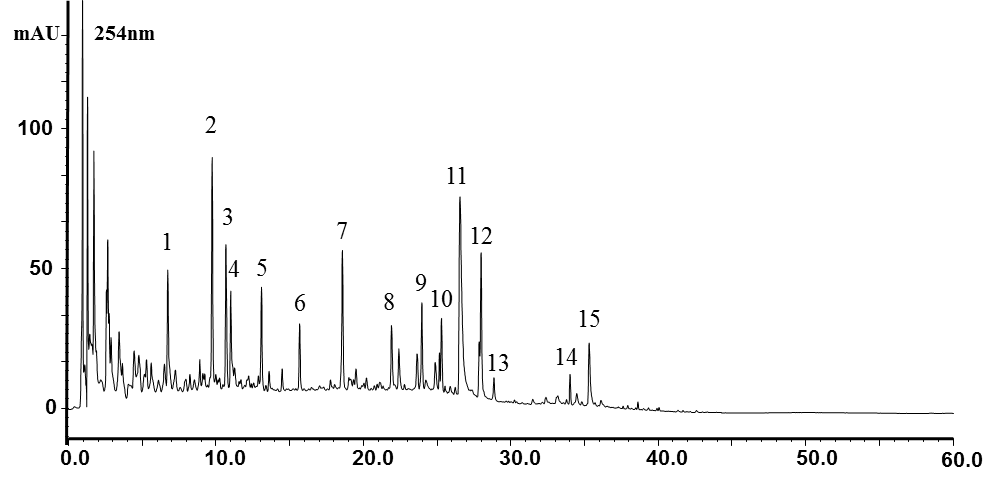


Fig 1. HPLC-PDA chromatogram of QSG at 254nm

1. Chlorogenic acid; 2. Cryptochlorogenin Acid; 3. Neochlorogenic acid; 4. Secologanic acid; 5. Sweroside; 6. Secoxyloganin; 7. Liquiritin; 8. Isochlorogenic acid A; 9. Isochlorogenic acid C; 10. Ononin; 11. Salvianolic acid B; 12. Calycosin; 13. Harpagoside; 14. Formononetin; 15. Glycyrrhizic Acid

B
