# Supplementary material for: Qishen Granule Improved Cardiac Remodeling via Balancing M1 and M2 Macrophages
Source: Front Pharmacol. 2019 Nov 25;10:1399. doi: 10.3389/fphar.2019.01399 (PMC6886583; doi:10.3389/fphar.2019.01399)

Figure S1: Panel 1 represents western blot shown in Figure 1C.


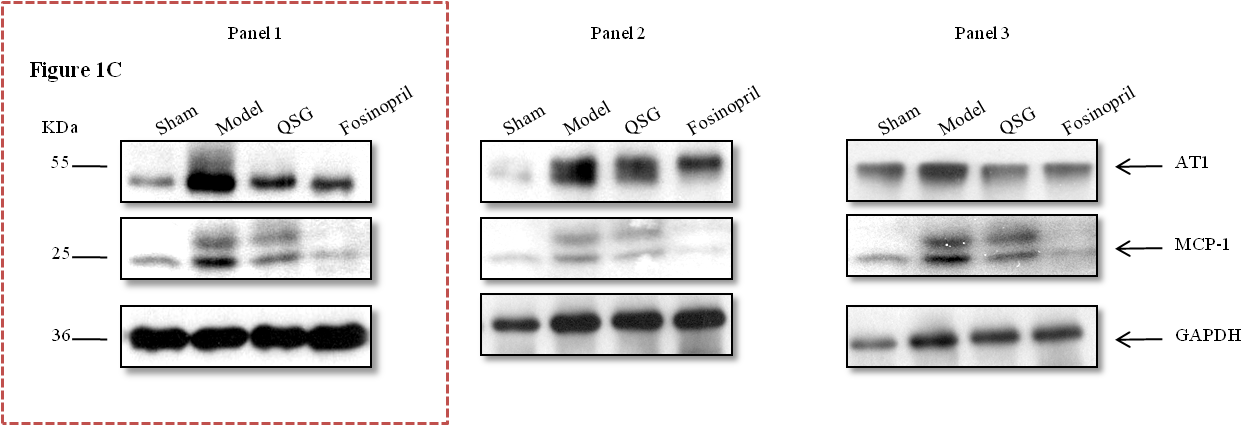


Figure S2: Panel 1 represents western blot shown in Figure 2C, D.


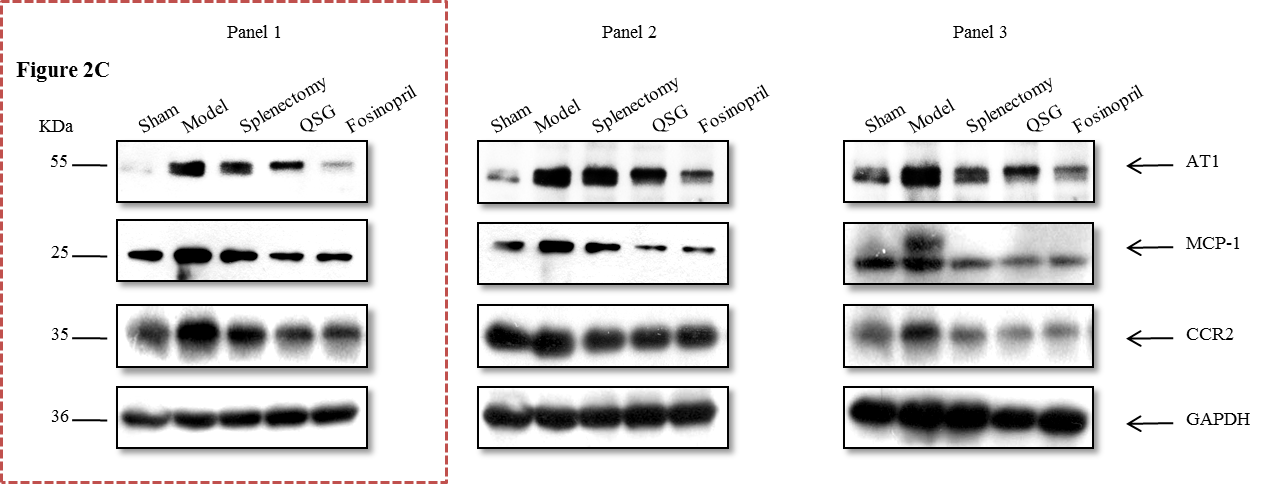


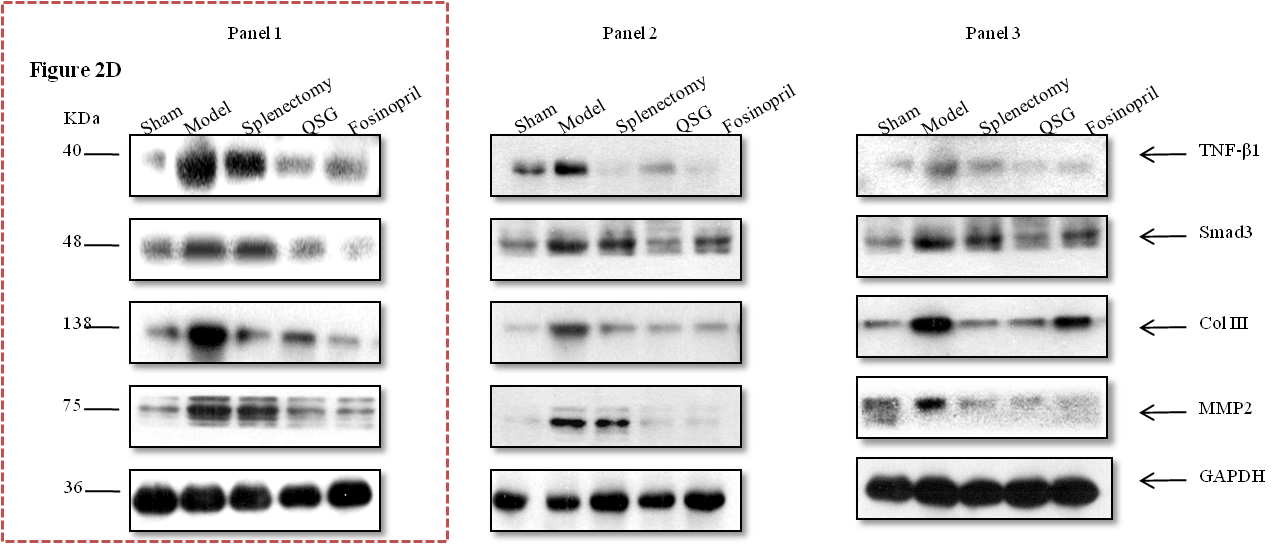


Figure S3: Panel 1 represents western blot shown in Figure 3C.


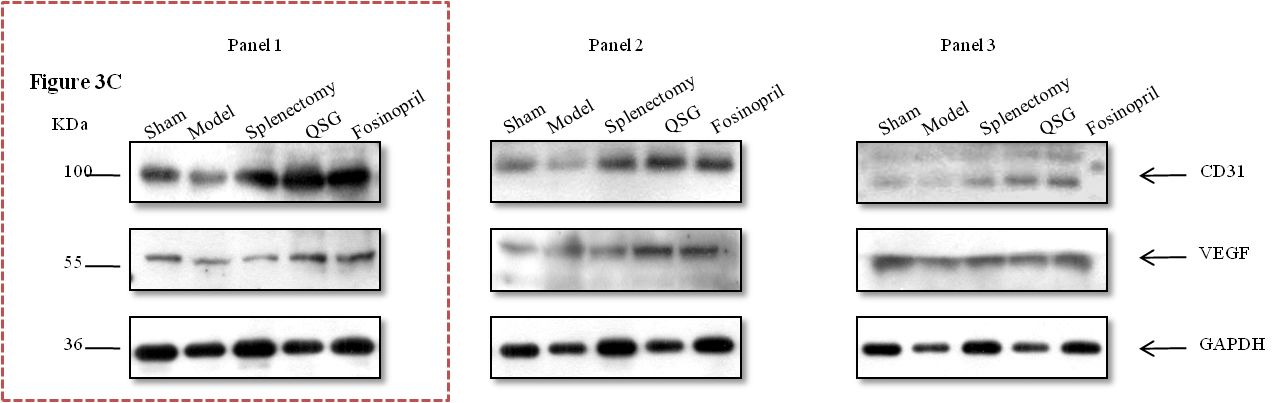

Supplement: Supplementary file 2 [file DataSheet_2.docx]
